# Supplementary material for: Causal Relationship between Aging and Anorexia Nervosa: A White-Matter-Microstructure-Mediated Mendelian Randomization Analysis
Source: Biomedicines. 2024 Aug 16;12(8):1874. doi: 10.3390/biomedicines12081874 (PMC11351342; doi:10.3390/biomedicines12081874)
Supplement: Supplementary file 1 [file biomedicines-12-01874-s001.zip › Supplementary Figure/Figure S7/Single SNP plot of MR analysis for ubm-b-1905 to AN.pdf]

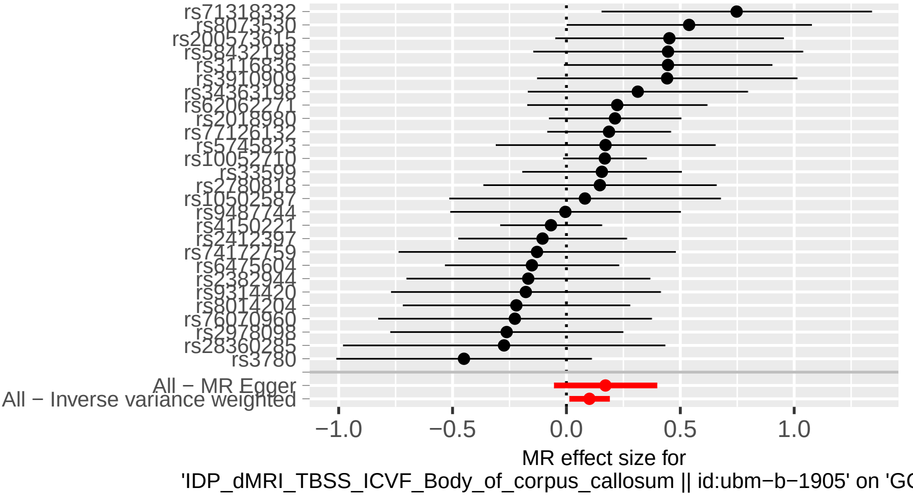

rs71318332
rs8073530
rs200573615
rs58432198
rs3116836
rs3910909
rs34363198
rs62062271
rs2018980
rs77126132
rs5745823
rs10052710
rs33599
rs2780818
rs10502587
rs9487744
rs4150221
rs2412397
rs74172759
rs6475604
rs2382944
rs9314420
rs8014204
rs76070960
rs2978098
rs28360285
rs3780
All − MR Egger
All − Inverse variance weighted
−1.0
−0.5
0.0
0.5
1.0
MR effect size for
'IDP_dMRI_TBSS_ICVF_Body_of_corpus_callosum || id:ubm−b−1905' on 'GC
